# Recombinase polymerase amplification (RPA) with lateral flow detection for three *Anaplasma* species of importance to livestock health

**DOI:** 10.1038/s41598-021-95402-y

**Published:** 2021-08-05

**Authors:** Andrea Salazar, Francisco M. Ochoa-Corona, Justin L. Talley, Bruce H. Noden

**Affiliations:** 1 Department of Entomology and Plant Pathology, Oklahoma State University, Stillwater, OK 74075 USA; 2 Institute of Biosecurity and Microbial Forensics, Oklahoma State University, Stillwater, OK 74075 USA

**Keywords:** Microbiology, Infectious-disease diagnostics

## Abstract

*Anaplasma marginale, A. ovis,* and *A. phagocytophilum* are the causative agents of bovine anaplasmosis, ovine anaplasmosis, and granulocytic anaplasmosis, respectively. The gold standard for diagnosis of post-acute and long-term persistent infections is the serological cELISA, which does not discriminate between *Anaplasma* species and requires highly equipped laboratories and trained personnel. This study addresses the development of a rapid, isothermal, sensitive, species-specific RPA assays to detect three *Anaplasma* species in blood and cELISA *A. marginale*-positive serum samples. Three RPA primer and probe sets were designed targeting *msp4* genes of each *Anaplasma* species and the internal control (GAPDH gene) for each assay. The limit of detection of gel-based or RPA-basic assays is 8.99 × 10^4^ copies/µl = *A. marginale*, 5.04 × 10^6^ copies/µl = *A. ovis*, and 4.58 × 10^3^ copies/µl = *A. phagocytophilum*, and for each multiplex lateral flow or RPA-*nfo* assays is 8.99 × 10^3^ copies/µl of *A. marginale,* 5.04 × 10^3^ copies/µl of *A. ovis*, 4.58 × 10^3^ copies/µl of *A. phagocytophilum,* and 5.51 × 10^3^ copies/µl of internal control (GAPDH). Although none of the 80 blood samples collected from Oklahoma cattle were positive, the RPA-*nfo* assays detected all *A. marginale* cattle blood samples with varying prevalence rates of infection, 83% of the 24 cELISA *A. marginale*-positive serum samples, and all *A. phagocytophilum* cell culture samples. Overall, although early detection of three *Anaplasma* species was not specifically addressed, the described RPA technique represents an improvement for detection of three *Anaplasma* in regions where access to laboratory equipment is limited.

## Introduction

Livestock production in the United States is a significant part of the economy. In 2017, cattle production was the most important livestock industry in the United States with an approximate value of $50.2 billon, followed by poultry ($42.7 billion) and swine production ($19.2 billion)^1^. One of the main tick-borne diseases impacting the cattle industry in the U.S. is bovine anaplasmosis which causes significant financial losses for producers^2, 3^. Increased mortality and morbidity due to bovine anaplasmosis affects the economy of the U.S. causing losses of more than $300 million per year while in Latin America, the losses are estimated to be approximately $800 million per year^3^.

Ticks are blood-sucking arthropods that transmit a wide variety of pathogens like viruses, bacteria, and protozoa^4^, including *Anaplasma* species. Anaplasmosis is caused by bacterium in the genus *Anaplasma* species and infects a broad range of animals such as cattle, sheep, and humans^5^. The three species of concern are *Anaplasma marginale, A. ovis*, and *A. phagocytophilum*. Detection methods for these pathogens include a wide variety of microscopy, antibody-based, and molecular methods^2, 6–8^. The current gold standard for *A. marginale* detection in cattle is a USDA-approved cELISA, a serologic test that targets a protein epitope of the highly conserved *Anaplasma msp5* gene; but it does not distinguish among species^6, 7^. A variety of polymerase chain reaction (PCR) assays exist that detect *Anaplasma* species but the low limit of detection for early and chronic infections, cost of the equipment needed to run the assay, and the need for trained personnel limits its effectiveness in point-of-care conditions^2, 9–12^.

Loop-mediated isothermal amplification (LAMP) and recombinase polymerase amplification (RPA) are isothermal methods that are being used to improve cost and time, and issues encountered by DNA-based diagnostics^13^. LAMP assays have been developed to detect *A. ovis, A. phagocytophilum*, and *A. marginale*^14–18^ but, to date, they are only being used in laboratory-based settings. Developed in 2006, RPA represents an innovative isothermal amplification technology that has been used to detect a range of pathogens in agriculture, human and veterinary medicine, as well as food safety^19, 20^. The advantage of RPA is the minimal investment for equipment due to low reaction temperatures (25–45 °C), relatively short incubation periods (20–40 min) and use of sensitive and specific primers and probe^20^. Additionally, RPA reagents can be stored for 3 weeks at room temperature without need for low-temperature storage because they are stable as lyophilized pellets^21^. Therefore, RPA can be applied directly in point-of-care diagnostic center or resource-limited areas while PCR or ELISA based analyses require expensive laboratory equipment and trained personnel.

Given the complex nature of *Anaplasma* infections and the current lack of point-of-care diagnostic tools, specific, sensitive, easy-to-use, and rapid isothermal detection assays are needed to improve the accuracy of *Anaplasma* diagnosis in livestock worldwide. The goal of this study was to develop and optimize multiplex lateral flow or RPA-*nfo* (exonuclase IV) (RPA TwistAmp® nfo) assays using DNA from experimental blood and serum samples infected with *A. marginale*, culture cells containing *A. phagocytophilum*, and *A. ovis* DNA which can be incorporated into a field-based testing protocol.

## Results

### RPA primer and probe design

The RPA primer sets and *nfo* probes have 100% identity and 100% query coverage to each *Anaplasma* accessions available in the GenBank nucleotide database (NCBI) (Table 1) using BLASTn. There were no matches detected among *Anaplasma* species with other bacteria. The in-silico specificity of GAPDH (internal control) RPA primer set and *nfo* probe showed 100% identity and 100% query coverage with mammalian species such as *Bos taurus* (cattle)*, Ovis aries* (sheep)*, Capra hircus* (goat)*,* and *Odocoileus virginianus* (deer) using BLASTn. No matches were detected among *Anaplasma* species.

**Table 1:** *Anaplasma marginale, A. ovis, A. phagocytophilum,* and GADPH gene (internal control) RPA primers and probes*.

Target Seq	Primer	Length	Tm	GC%	Product size (bp)	Sequence
*A. marginale*	Am3L_msp4	33	76.63	54.55	103	ACGAAGTGGCTTCTGAAGGGGGAGTAATGGGAG
	Am3R_msp4	30	72.99	50		[5′Label-Biotin] GACTCACGCATGTCGAACGAGGTAACAGAA
	Am3_probe	39	70.4	56.4		[5′-Label-FAM] TAGCTTTTACGTGGGTGCGGCCT[THF]CAGCCCAGCATTTCC[3′-block-C3spacer]
*A. phagocytophilum*	ApL_msp4	30	75.14	60	202	TGCGGCCGCAGTATGTGCCTGCTCCCTTTT
	ApR_msp4	30	62.31	40		[5′Label-Biotin] GTTATAACCTTTTACGTAAGATCCTCCCCT
	Ap_probe	52	68.2	44.2		[5′Label-FAM] TGATGCGTCTGATGTTAGCGGTGTTATGAACGG[THF]AGCTTTTACGTAAGTGGT [3′block-C3spacer]
*A. ovis*	Ao2L_msp4	35	67.75	45.71	184	AGAGACCTCGTATGTTAGAGGCTATGACAAGAGTG
	Ao2R_msp4	35	68.57	48.57		[5′Label-Biotin] CCTTCTGTAGCTTGCTTCTAGTTCCACTCTAGCTC
	Ao2_probe	50	65.2	38		[5′Label-FAM] AAATCCGGCTACACTTTTGCTTTCTCTAAGAA[THF]TTACTCACATCTTTCGA [3′block-C3spacer]
*GAPDH gene*	GAPDH2_L	30	72.53	50	168	CTGAGACAAGATGGTGAAGGTCGGAGTGAA
	GAPDH2_R	27	72.82	51.85		[5′Label-Biotin] TTGCCGTGGGTGGAATCATACTGGAAC
	GAPDH_Probe	51	70.3	51		[5′Label-DIG] CTGGCAAAGTGGACATCGTCGCCATCAATGACCC[THF]TTCATTGACCTTCACT[3′block-C3spacer]

*Primers, probes, and the amplification kit are protected by a patent application.

### Artificial positive control (APC)

Endpoint PCR was used to confirm the size of the two complete APC sequences. The PCR product size of the RPA-basic APC was 250 bp (from *A. phagocytophilum* forward (Ap_L) to GAPDH reverse (GAPDH2_R) primers, Supplemental Fig. 1A) and the RPA-*nfo* for lateral flow APC, which was 442 bp (from *A. phagocytophilum* forward (Ap_L) primer to *A. ovis* reverse (Ao2_R) primer, Supplemental Fig. 1B). Furthermore, all targets of both APCs were amplified individually using RPA-basic reactions. All the resulting amplicons were detected by agarose gel electrophoresis. The product size between APCs and actual *Anaplasma* reference controls were slightly different as expected. The RPA amplicons amplified using the RPA-basic_APC with primers for *A. marginale* (Am3R/L), *A. ovis* (Ao2R/L), *A. phagocytophilum* (ApR/L), and GAPDH (GAPDH2R/L) were 93 bp, 193 bp, 188 bp, and 152 bp, respectively; while the products amplified using reference *A. marginale, A. ovis, A. phagocytophilum,* and GAPDH DNA were 103 bp, 184 bp, 202 bp, and 168 bp, respectively. The expected RPA product size of the RPA-*nfo*_APC using this synthetic control were 137 bp, 189 bp, 220 bp, and 160 bp which were amplified using *A. marginale* primers (Am3R/L), *A. ovis* primers (Ao2R/L), *A. phagocytophilum* primers (ApR/L), and GAPDH primers (GAPDH2R/L), respectively.

### Optimization of RPA-basic and RPA-*nfo* conditions

 Betaine, temperature, and incubation time were optimized using RPA-basic reactions based on agarose gel results. Betaine (10 µl) was added to the RPA reaction to minimize false-positives and to reduce mis-priming. Agarose gel results confirmed that non-template controls did not amplify non-specific targets when betaine was added to the reaction nor did it interfere with the amplification of RPA targets. The three primer sets amplified the three expected diagnostic products from the predicted *msp4* gene of *A. marginale* (103 bp)*, A. ovis* (184 bp), and *A. phagocytophilum* (202 bp) within a range of six temperatures (35–40 °C). The reaction times were 20 or 40 min, indicating adequate performance in a broad range of temperatures and reaction times. The band intensity was the same for each assay so 37 °C with a reaction time of 20 min were selected for routine assay. None of the *Anaplasma* species primer sets amplified non-specific products from the non-template control (water).

The amplified RPA-*nfo* products of *A. marginale, A. ovis,* and *A. phagocytophilum* were detected in lateral flow assay (LFA). The presence of control line C confirmed the lateral flow assay was working properly, test line 1 showed the internal control (GADPH gene), and test line 2 verified the presence of three *Anaplasma* species in the samples. All tests were consistent for each repeated assay demonstrating consistency in the amplification of all bacterial targets tested, only the control line C developed in the negative non-template control (water).

Faint test lines were observed when the incubation time was 5 min using lateral flow assay. However, as the incubation time was increased to 10 min and 15 min, stronger positive signals were detected. Based on these results, the RPA-*nfo* reaction time consisted of two-steps: first, RPA amplification in dry bath incubator at 37 °C for 20 min, and second, lateral flow assay at room temperature for 10 min as recommended for a total incubation time of 30 min.

RPA-basic reactions were performed using total DNA extracted from *A. marginale*-infected cattle blood and sheep blood spiked with *A. ovis* DNA*.* The RPA primer set GAPDH2R/L (internal control) amplified a product from the predicted target of the GAPDH gene at 37 °C for 20 min. The RPA products were obtained within the expected amplification size of 168 bp. The reverse and forward RPA primers did not amplify products from the negative control tick DNA and the non-template control (water).

Multiplex RPA-*nfo* reactions targeted each *Anaplasma* species with the GAPDH housekeeping gene (internal control). The results of sixteen multiplex RPA-*nfo* assays using an Artificial Positive Control (APC: 1 ng/µl) as a template demonstrated *A. marginale* and internal control test lines were clear and intense when primers were loaded at a volume of 1.8 µl (0.36 µM final concentration) and the probes 0.2 µl (0.04 µM final concentration). The best combination for *A. ovis* and *A. phagocytophilum* was 1.05 µl of primers (0.21 µM final concentration) and 0.6 µl of probes (0.12 µM final concentration). In these assays, the two test bands (line 1 and 2) were equally intense and clear*.* No signal was observed in non-template control (water).

### Specificity of RPA-basic and RPA-*nfo* assays

**Figure 1:**
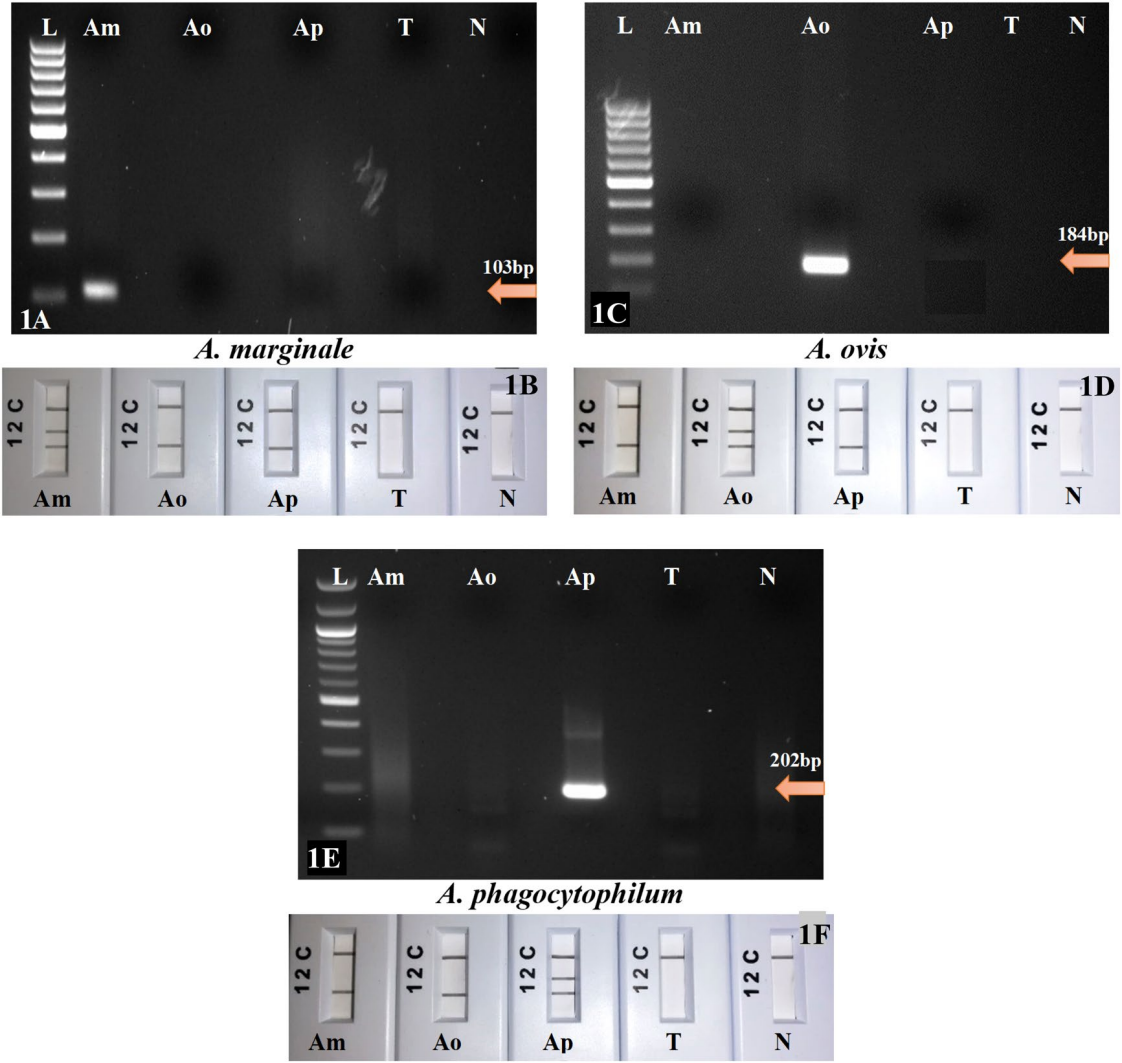
Specificity assay among *Anaplasma* species electrophoresed products obtained by RPA-basic primers in the top panel and the multiplex RPA-*nfo* reactions in corresponding PCRD lateral flow devices in the bottom panel. (**A**) Gel-based RPA primers. Lane L, 100 bp DNA ladder; lane Am, *A. marginale;* lane Ao, *A. ovis;* lane Ap, *A. phagocytophilum;* lane T, lab-reared tick DNA; lane N, non-template control (NTC, water). (**B**) Multiplex RPA-*nfo* primers and probes. Lane C, flow-check line; Lane 2, detects FAM/Biotin labelled amplicons (*Anaplasma* species); Lane 1, detects DIG/Biotin labelled amplicons (GAPDH—internal control); Am, *A. marginale;* Ao, *A. ovis;* Ap, *A. phagocytophilum;* T, lab-reared tick DNA; N, non-template control (water).

Each species-specific primer set for *A. marginale*, *A. ovis*, and *A. phagocytophilum* amplified the specific sample DNA and no amplification was observed when primers were tested against the other two *Anaplasma* species (Fig. 1A,C,E). No cross-amplification was observed in any reaction. Lab-reared tick DNA was used as negative control and NTC (no template control) was also included in each of the tests. These negative controls did not produce any reaction using the RPA-basic reactions.

The multiplex RPA-*nfo* tested positive only in each of the *Anaplasma* species target; a solid positive test internal control band developed in each of the *Anaplasma* species DNA, whereas no signals (lines 1 and 2) were observed in the negative dipsticks (tick and NTC) (Fig. 1B,D,F). The results indicated that the primer–probe combinations designed for RPA-*nfo* reactions were specific to each of the corresponding *Anaplasma* species targets. The species-specific isothermal reactions consistently detected and discriminated the three *Anaplasma* species.

### Limit of detection of RPA-basic and RPA-*nfo* assay with plasmids and infected samples

**Figure 2:**
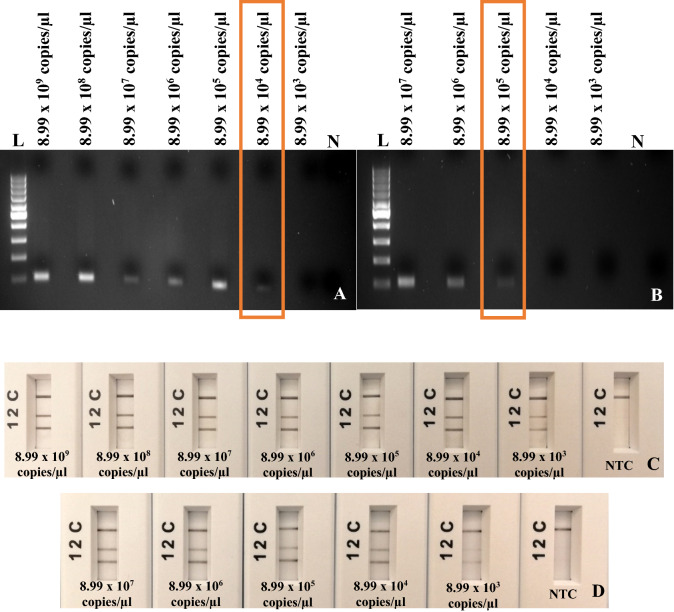
Limit of detection assays of *A. marginale.* (**A**) RPA-basic primers using ten-fold serial dilution of *A. marginale* plasmid from 8.99 × 10^9^ to 8.99 × 10^3^ copies/µl. (**B**) RPA-basic primers using Ten-fold serial dilution of *A. marginale* total DNA from 8.99 × 10^7^ to 8.99 × 10^3^ copies/µl. Lane N, non-template control (NTC, water); lane L, 100 bp DNA ladder. (**C**) Multiplex RPA-*nfo* primers and probe using ten-fold serial dilution of Artificial Positive Control (APC). (**D**) Multiplex RPA-*nfo* primers and probes using ten-fold serial dilution of *A. marginale* total DNA from 8.99 × 10^7^ to 8.99 × 10^3^ copies/µl. Lane C, flow-check line; Lane 2, detects FAM/Biotin labelled amplicons (*A. marginale*); Lane 1, detects DIG/Biotin labelled amplicons (GAPDH—internal control); Lane N, non-template control (water).

**Figure 3:**
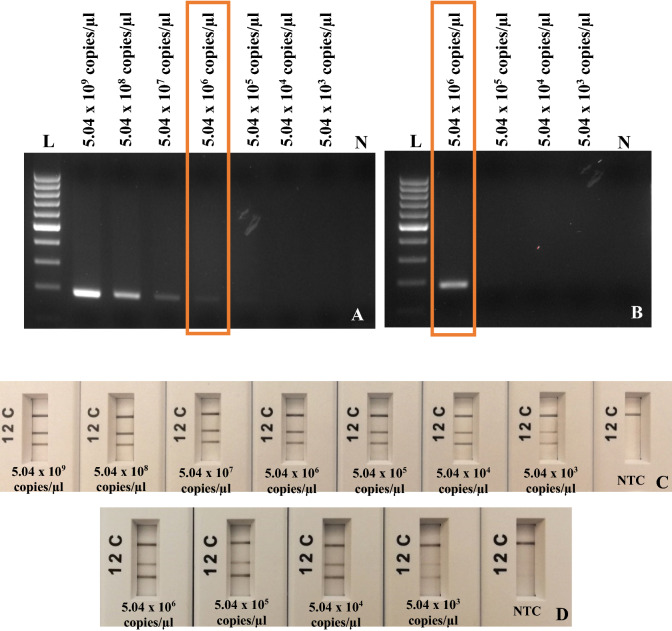
Limit of detection assays of *A. ovis*. (**A**) RPA-basic primers with ten-fold serial dilution of *A. ovis* plasmid from 5.04 × 10^9^ to 5.04 × 10^3^ copies/µl. (**B**) RPA-basic primers with ten-fold serial dilution of *A. ovis* total DNA from 5.04 × 10^6^ to 5.04 × 10^3^ copies/µl. Lane N, non-template control (NTC, water); lane L, 100 bp DNA ladder. (**C**) Multiplex RPA-*nfo* primers and probes using ten-fold serial dilution of Artificial Positive Control (APC). (**D**) Multiplex RPA-*nfo* primers and probes using ten-fold serial dilution of *A. ovis* total DNA from 5.04 × 10^6^ to 5.04 × 10^3^ copies/µl. Lane C, flow-check line; Lane 2, detects FAM/Biotin labelled amplicons (*A. ovis*); Lane 1, detects DIG/Biotin labelled amplicons (GAPDH—internal control); Lane N, non-template control (water).

**Figure 4:**
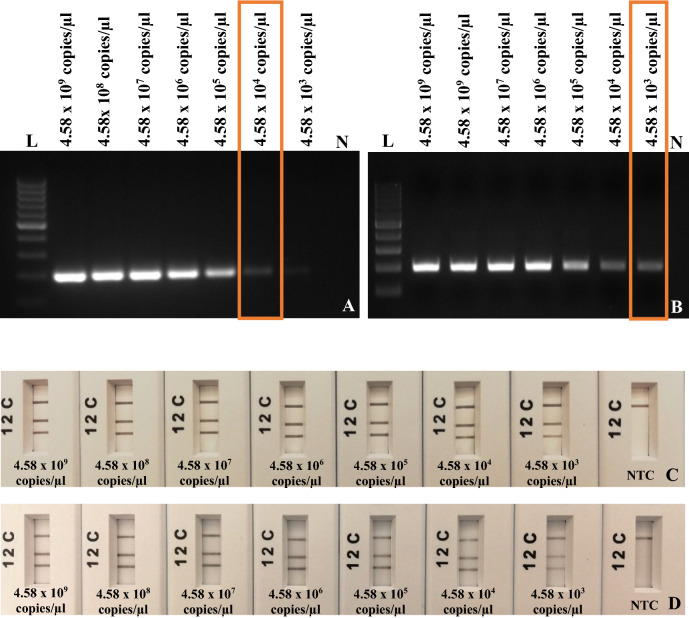
Limit of detection assays of *A. phagocytophilum*. (**A**) RPA-basic primers using ten-fold serial dilution of *A. phagocytophilum* plasmid from 4.58 × 10^9^ to 4.58 × 10^3^ copies/µl. (**B**) RPA-basic primers using ten-fold serial dilution of *A. phagocytophilum* total DNA from 4.58 × 10^9^ to 4.58 × 10^3^ copies/µl. Lane N, non-template control (NTC, water); lane L, 100 bp DNA ladder. (**C**) Multiplex RPA-*nfo* primers and probe using ten-fold serial dilution of Artificial Positive Control (APC). (**D**) Multiplex RPA-*nfo* primers and probe using ten-fold serial dilution of *A. phagocytophilum* total DNA from 4.58 × 10^9^ to 4.58 × 10^3^ copies/µl. Lane C, flow-check line; Lane 2, detects FAM/Biotin labelled amplicons (*A. phagocytophilum*); Lane 1, detects DIG/Biotin labelled amplicons (GAPDH—internal control); Lane N, non-template control (water).

The purified plasmid of *A. marginale* (8.99 × 10^9^–8.99 × 10^3^ copies/µl)*, A. ovis* (5.04 × 10^9^–5.04 × 10^3^ copies/µl), and *A. phagocytophilum* (4.58 × 10^9^–4.58 × 10^3^ copies/µl) by serial dilution was used for limit of detection of the RPA-basic method. The results demonstrated that limit of detection on number of copies per µl of RPA-basic *A. marginale* was 8.99 × 10^4^ copies/µl, *A. ovis* was 5.04 × 10^6^ copies/µl, and *A. phagocytophilum* was 4.58 × 10^2^ copies/µl (Figs. 2A, 3A, 4A).

A quantitative PCR standard curve was used to determine the concentration of *A. marginale, A. ovis,* and *A. phagocytophilum* in total extracted DNA. The concentration of bacteria in the samples was 9.21 × 10^7^ copies/µl for *A. marginale:*, 6.3 × 10^6^ copies/µl for *A. ovis:*, and 6 × 10^10^ copies/µl for *A. phagocytophilum*: Therefore, these three samples were used in RPA-basic and RPA-*nfo* limit of detection assays. Additionally, the three quantitative PCR showed a similar significant tendency (r = 0.99).

*A. marginale* quantified sample was diluted from 8.99 × 10^9^ to 8.99 × 10^3^ copies/µl, the limit of detection of RPA-basic using total DNA was 8.99 × 10^3^ copies/µl (Fig. 2B). The limit of detection of RPA-basic to detect *A. marginale* from plasmid DNA was 10 times more sensitive than from an infected blood sample. The *A. ovis* quantified sample was diluted from 5.04 × 10^6^ to 5.04 × 10^3^ copies/µl, the limit of detection of RPA-basic using total DNA was 5.04 × 10^6^ copies/µl (Fig. 3B). The *A. phagocytophilum* quantified sample was diluted from 4.58 × 10^9^ to 4.58 × 10^3^ copies/µl, the limit of detection of RPA-basic using total DNA was 4.58 × 10^3^ copies/µl (Fig. 4B). The limit of detection of RPA-basic to detect *A. phagocytophilum* and *A. ovis* from plasmid DNA and an extracted total DNA sample was equivalent. No amplification was observed with non-template control in each.

The limit of detection of RPA-*nfo* assays was measured using a ten-fold serial dilution of the Artificial Positive Control (APC). The results of *A. marginale, A. ovis,* and *A. phagocytophilum* RPA reactions using primers and probes shown that method allows detecting as low as was 8.99 × 10^3^ copies/µl of *A. marginale,* 5.04 × 10^3^ copies/µl of *A. ovis*, 4.59 × 10^3^ copies/µl of *A. phagocytophilum,* and 5.51 × 10^3^ copies/µl of internal control (GAPDH) with APC and bacterial measured total DNA (Figs. 2C,D, 3C,D, 4C,D). Clear test (1, 2) and control lines appeared on each strip; however, 1 and 2 test lines were faint when DNA concentration was decreasing. Only the control line band was observed with non-template control (water) in each assay. Therefore, the limit of detection of RPA-*nfo* was higher than the RPA-basic amplification detected by agarose gel electrophoresis.

### End-point PCR and RPA analyses using serum and blood samples

**Figure 5:**
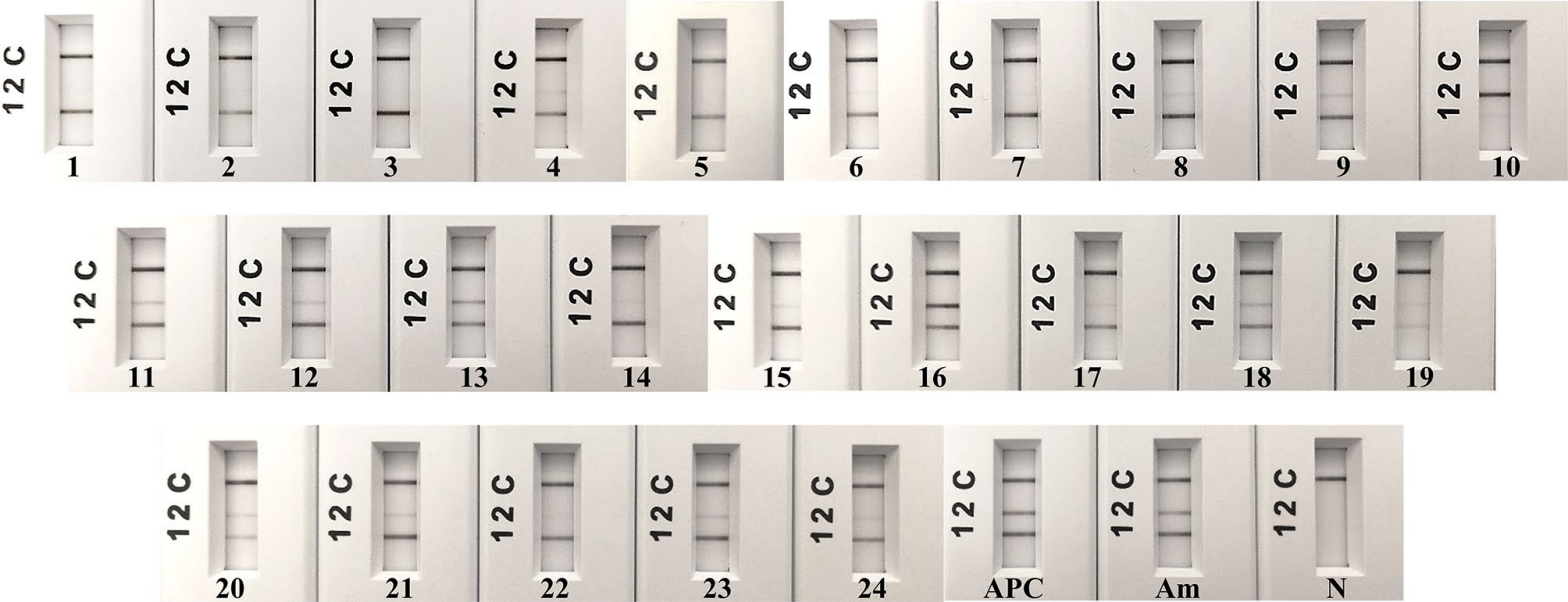
*A. marginale* multiplex RPA-*nfo* using positive serum samples. Lane C, flow-check line; Lane 2, detects FAM/Biotin labelled amplicons (*A. marginale)*; Lane 1, detects DIG/Biotin labelled amplicons (GAPDH gene); 1–24, *A. marginale* positive serum samples; APC, Artificial positive control; Am, *A. marginale* positive reference control; NTC, non-template control (water).

Twenty-four cELISA *A. marginale*-positive serum samples were simultaneously detected by endpoint PCR and multiplex RPA-*nfo*. Out of the 24 samples, only one (4.2%) tested positive by endpoint PCR while 20 samples (83.3%) tested positive by *A. marginale* multiplex RPA-*nfo* (Supplemental Table 1 and Fig. 5). The internal control test line (1) appeared in each serum sample. *A. marginale* test lines (2) were faint and not clear in four serum samples (Samples 4, 6, 7, 14. Fig. 5). No amplification was observed with non-template control (water) of both assays. Expected PCR product size of 344 bp was visible with *A. marginale* reference positive control. Two test lines (1, 2) were observed with APC and *A. marginale* reference positive control in PCRD cassette (Fig. 5).

Twenty-five *A. marginale* positive blood samples and three *A. phagocytophilum* positive cell culture samples were simultaneously detected by qPCR using optimized RPA primers and multiplex RPA-*nfo* (Supplemental Table 2, Supplemental Figs. 2 and 3). Out of 25 samples of stored bovine blood containing varying infection levels and *A. marginale* isolates, 24 (96%) were positive for *A. marginale* by both diagnostic techniques (Supplemental Table 2 and Supplemental Fig. 2). While the amount of quantified *Anaplasma* DNA in total extracted blood DNA may have varied between blood samples and infection levels due to unspecified methodologies in the source lab, the RPA assay still detected *A. marginale* DNA in all of them (Supplemental Table 1). All cell culture samples tested positive for *A. phagocytophilum* by qPCR and RPA-*nfo* reactions (Supplemental Table 2 and Supplemental Fig. 3). No amplification was observed with non-template control (water) of both assays. Two test lines (1, 2) were observed with APC, *A. marginale,* and *A. phagocytophilum* reference positive control in PCRD cassette.

All 80 field-collected blood samples tested negative to *A. marginale* by endpoint PCR. To preserve the use of lateral flow assay, two randomly selected DNA blood samples of each sale barn were assayed using RPA-basic and RPA-*nfo*. All eight samples tested negative for *A. marginale*. Expected PCR and RPA product sizes of 344 bp and 103 bp were visible with *A. marginale* reference positive control, respectively. Two test lines (1, 2) were observed with APC and *A. marginale* reference positive control in lateral flow assay and no amplification was observed with non-template control (water) of the three assays.

## Discussion

Rapid diagnostic tests are useful for screening livestock at point-of-care must not only be able to detect low parasitemias of *Anaplasma* infections in chronically-infected animals, but must also be simple to use by untrained personnel. This feature involves the use of no expensive equipment and easy to interpretation^22^. This study describes the development of three RPA primer and probe sets for rapid, sensitive, and species-specific detection of *A. marginale, A. ovis,* and *A. phagocytophilum* by RPA-basic and multiplex RPA-*nfo* using GAPDH gene as internal control. Coupling RPA-*nfo* assays in a lateral flow assay creates the opportunity to develop rapid point-of-care diagnostic tests for three *Anaplasma* species which affect cattle, sheep, and goats. When put together with an Elution Independent Collection Device (EICD) prototype^23^, the whole process from blood extraction to accurate detection of species-specific *Anaplasma* species at the point of sampling is 60–70 min.

Although 2019 was a low year for *Anaplasma* infections in Oklahoma cattle (Justin Talley, personal communication), the usefulness of the rapid Anaplasma detection (RAD) assay was demonstrated in the detection of 83% of the cELISA *A. marginale*-positive serum samples from cattle compared with only 4% by endpoint PCR. These results are due to the dramatic differences in limit of detection between the multiplex RPA-*nfo* assay which detects at 8.99 × 10^4^ copies/µl while the limit of detection of the endpoint PCR is 1.35 × 10^6^ copies/µl. In addition to cELISA *A. marginale*-positive serum samples, the *A. marginale* RAD assay detected 96% of varying *A. marginale* infections in stored positive cattle blood samples and the *A. phagocytophilum* RAD assay detected all cell culture samples. These *A. marginale* infections consisted of chronic infections in the same cattle—one of which was undetectable by microscopy—as well as two different isolates. Together with the detection of *A. marginale* DNA in cELISA *A. marginale*-positive serum samples, which is the industry standard, this demonstrates the effectiveness and high limit of detection of our RPA-*nfo* assay over PCR detecting low infection rates of *Anaplasma* in cattle. While single-use RPA assays have been developed to diagnose tick-borne diseases such as Rocky Mountain spotted fever^24^, theileriosis^25, 26^, Lyme borreliosis^27^, equine piroplasmosis^28^, Crimean-Congo Hemorrhagic fever ^29^ and *A. phagocytophilum*^30, 31^, there are no reported studies for *A. marginale* and *A. ovis* detection.

Sensitive diagnostics are used to detect *Anaplasma* infections in livestock in diagnostic labs worldwide^10, 11^, but there is a need for point-of-care diagnostics for the detection of *Anaplasma* that will screen livestock in field-conditions. The species focused on in this study are important globally. *Anaplasma marginale* is a problem in the US, not only in the states where most livestock is raised but also in other states as well^32–37^, in addition to cattle and goats worldwide^38–42^. While *A. ovis* has not been reported to adversely affect sheep and goats, it continues to be identified in ovine and caprine species, domestic and wild, worldwide^43, 44^. The strains of *A. phagocytophilum* in the United States do not appear to infect bovine populations^23, 45^ but they do cause death and morbidity in European cattle^46, 47^. One of the issues that is often reported for the standard test used in professional diagnostic labs is that the cELISA does not discriminate between *Anaplasma* species as it is based on the *msp5* gene which is cross-reactive among *Anaplasma* species^2, 48^. In the current assay, each RPA primer/probe set was based on the *msp4* gene which specifically differentiates between the species and ensures that each assay will only detect one species of *Anaplasma*. In addition to being based on a species-specific gene, we recognized the many strains of *A. phagocytophilum* worldwide, some affecting humans while some affect cattle^7, 49^. Our *A. phagocytophilum* RPA primer/probe set was developed from over 20 different strains to ensure the detection of the majority of strains currently reported. While our assay is built for consensus detection, it would be possible to build strain specific primer/probe sets as well. Altogether, there is still much to learn about the different strains of these pathogens and how they affect livestock species in different countries. As these studies continue to reveal the extent to which *A. marginale*, *A. ovis*, and *A. phagocytophilum* strains are impacting livestock development globally, the need for point-of-care detection of these pathogens at the local, community level becomes ever more important.

RPA is a relatively new technology, so we identified some key aspects that are imperative when developing sensitive and specific RPA primers and probes. First, development of accurate RPA reactions requires primers (30–35 nucleotides) and probes (46–52 nucleotides) which are longer than conventional PCR primers, however there are no optimal software packages from which to develop the RPA primers. We designed the primers using the web interface application Primer3^50^ and manually created the RPA probes according to the selection parameters for the optimal RPA primers and probes described in TwistAmp Design Manual^51^. As RPA product size influences the quality of bands, the best RPA primers were *A. ovis* and *A. phagocytophilum,* which generated intense and clear bands (more than 150 bp) compared with bands generated by RPA primers were *A. marginale* and GAPDH internal control. As the variation was not clear, further studies focused on primer and probe parameters are needed to improve band intensity of *A. marginale*. Non-specific amplification was eliminated by using betaine which is used in PCR, LAMP, and RPA to prevent secondary structures due to high GC content (varying between 40 and 60%) in target sequences, primers, and probes which may favor hairpin formation and create false-positive results^52^. The addition of betaine allowed positive results at a wider range of temperatures (35–40 °C). Finally, while commonly reported for LAMP assays^53, 54^, inactivation of RPA reactions at 80 °C for 5 min prevented possible cross-contamination among reactions.

To develop multiplex RPA-*nfo* assays, we optimized the multiplex RPA primers and probes to avoid cross-interaction between dyes and primer/probe secondary structure or hairpins formation which cause lower signal intensity in the test lines or false-positive results, respectively^55, 56^. In this study, two targets, *A. marginale msp4* and GAPDH genes, *A. ovis msp4* and GAPDH genes, and *A. phagocytophilum msp4* and GAPDH genes, were tested in single multiplex RPA-*nfo* reaction with optimized primer and probe combinations. In total, the amplifications obtained two clear, intense lines on each reaction unit which could be easily read in any light. In the future, it would be possible to place all three reactions with their controls into one unit as some commercial serological tests are currently packaged.

One of the challenging aspects of developing molecular diagnostics is the need for target pathogen DNA on which to validate that the assays are detecting the pathogen and reducing the changes of false-negative results. Positive controls are often difficult to obtain because not all proteins or pathogens are available, or some pathogens are exotic and not commercially available^57^. At times, samples containing infectious material are also used as positive controls, however, shipping and handling of these samples is risky and require permits^58^. The development of an artificial positive control (APC) involves the use of customized synthetic DNA inserts based on linear arrays of primer sequences designed from a variety of organisms or targets important in detection, diagnostics or research^59^. Used previously to mimic multiple pathogens^22^, APCs can reduce risks associated with in vivo positive controls and improve accuracy of molecular detection techniques. In the current study, we used two constructed synthetic DNA positive controls targeting *A. marginale*, *A. ovis*, *A. phagocytophilum*, and GAPDH housekeeping genes using species-specific RPA primers and *nfo* probes synthesized in a pUC57 vector system. When these two APCs were compared with *Anaplasma*-infected blood samples as reference positive controls in the RPA-basic and RPA-*nfo* assay systems, we found they both demonstrated accurate amplification. The amplicons generated by the RPA assay varied slightly in size from those generated by the *Anaplasma* reference positive controls. This was most likely due to differing annealing sites of target sequences in vivo and the distribution of primers and probe sequences in APC. However, these variations were not an issue because the *Anaplasma* species and internal control targets amplified as well demonstrating that APCs worked correctly.

## Conclusion

The *Anaplasma*-specific RPA assays developed in this study are part of a wider project to develop rapid diagnostic assays that can detect the extremely low parasitemia levels of *Anaplasma* infections in chronically infected animals. To augment this goal, the RPA assays were developed in conjunction with a lateral flow assay to make the technique simple to use at a point-of-sampling site, the results easy to interpret, and able to be used by untrained personnel. When put together with an Elution Independent Collection Device (EICD) prototype^23^, the whole process from blood extraction to accurate detection of species-specific *Anaplasma* took 60–70 min. While this process continues to be streamlined to reduce the testing period, this marks an important step in the development of point-of-care diagnostics for *Anaplasma* species which can be used by field-based veterinarians as well as APHIS agents to monitor livestock at ports of entry into the United States. This becomes even more important as co-infections of two or more *Anaplasma* species have been reported in ticks, deer, and cattle in different parts of the world^2, 60^. In this study, specificity assays of each set of RPA primers and probes as well as the multiplex RPA-*nfo* reactions detected only the specific *Anaplasma* sample targets. Both RPA-basic and multiplex RPA-*nfo* identified and discriminated among three *Anaplasma* species and detected *Anaplasma marginale* DNA in the serum of 83% of cELISA *A. marginale*-positive cattle and 96% of *A. marginale* positive blood samples. By combining these species-specific RPA assays for three *Anaplasma* species with appropriate controls in a lateral-flow delivery system, we have demonstrated the flexibility and utility of this molecular technique in the development of many types of field-diagnostics.

## Materials and methods

### Source of samples

Reference positive controls consisting of frozen bovine blood infected with *Anaplasma marginale,* ovine blood infected with *A. ovis*, and cultured cells containing *A. phagocytophilum* in DMSO were provided by the Oklahoma State University College of Veterinary Medicine, Stillwater, USA (Dr. Kathy Kocan). The *A. marginale* bovine samples were from long-term acute and chronic infection studies which tracked blood prevalence rates in the same cattle over time. Most isolates were derived from Oklahoma samples, but one was from Virginia and another from *Dermacentor albipictus* ticks. Lyophilized *A. ovis* DNA was provided by Instituto de Investigación en Recursos Cinegéticos—Sabio, Spain (Dr. José de la Fuente). Lab-reared ticks (*Dermacentor variabilis*) provided by the OSU Tick-Rearing Facility (Stillwater, USA) were used as negative control in the assays. *A. marginale* cELISA-positive serum samples from Oklahoma-based cattle were provided by Oklahoma State University College of Veterinary Medicine, Stillwater, USA (Dr. Jerry Saliki). Dimethyl sulfoxide (DMSO) was removed from *A. marginale* experimental blood isolates by incubating the samples at 55 °C for 5 min and centrifuging for 15 min at 8000 rpm. The supernatant was discarded, 200 μl of 1 × Phosphate Buffer Saline (PBS) was added to the pellet, mixed thoroughly and vortexed (Dr. Kathy Kocan, personal communication).

Additionally, blood was collected from 80 randomly chosen cattle at 4 different livestock auctions sites in Oklahoma (20 cattle per auction site) during August and September 2019, the time most likely to encounter *Anaplasma*-infected cattle in Oklahoma^37^. The blood was collected by an authorized veterinarian using purple-top tube (Fisher Scientific) under the auspices of an Animal Care and Use Protocol (AG-18-3) approved by the IACUC at Oklahoma State University. The cattle selected originated from 20 Oklahoma counties and included one county in Texas and another in Tennessee. Collected blood was stored at 4 °C until DNA extraction. Total DNA from blood samples, infected cell cultures, and lab-reared tick (*Dermacentor variabilis*) was extracted using QIAmp Blood Mini kit (Qiagen, USA) following the manufacturer’s instructions.

### RPA primer and probe design

The major surface protein 4 genes (*msp4*) sequences of *A. ovis* strain MD2059 (Accession number: DQ674249.1) and *A. marginale* Brazil isolate (Accession number: AY714546.1) were aligned, focusing on a region where the two species showed a major difference. The *A. marginale msp4* sequence had a deletion of three nucleotides not traceable in the *A. ovis msp4* gene, which was found at nucleotide position 120 of accession AY714546.1. The RPA primers were designed from nucleotide position 101 to 131 and 174 to 203 of the *A. marginale msp4* sequence, respectively. The *A. ovis* RPA primers and *nfo* probe were designed from a consensus sequence of twelve *msp4* gene sequences (NCBI accession numbers: FJ460455.1, FJ460454.1, FJ460453.1, FJ460452.1, FJ460451.1, FJ460450.1, FJ460449.1, FJ460448.1, FJ460447.1, FJ460446.1, FJ460445.1, FJ460444.1). Primers and probe were located between nucleotides 213–396.

Due to high variability among *A. phagocytophilum* strains, it was not possible to calculate a consensus sequence; however, a phylogenetic tree was generated using all the available *msp4* gene from NCBI to select the closely related sequences. Twenty-six sequences from NCBI (Accession number: KC847317.1, KP861635.1, KP861634.1, AY706390.1, AY706389.1, AY706388.1, AY706387.1, AY702925.1, MF974855.1, MF974854.1, HQ661163.1, HQ661162.1, HQ661159.1, HQ661158.1, HQ661157.1, HQ661156.1, HQ661155.1, HQ661154.1, AY829456.1, AY829455.1, AY530198.1, AY530197.1, AY530196.1, AY530195.1, AY530194.1, JQ522935.1) were aligned using ClustalX, and an RPA primer set and *nfo* probe were designed based on the consensus sequence. The internal control RPA primer set and RPA-*nfo* probe were designed from the glyceraldehyde 3-phosphate dehydrogenase gene (GAPDH) from three *Bos taurus* sequences (NCBI accession numbers: NM 001034034.2, BC102589.1, XM_027541122.1) at nucleotide position 86–253. The glyceraldehyde 3-phosphate dehydrogenase (GAPDH) gene is a conserved gene found in mammalian cells and has been used extensively as internal control for detection methods^61^.

RPA primers were designed using Primer3^50^ while the thermodynamics and tendency to form self-dimers was analyzed using mFold^62^. The selected parameters for optimal RPA primers were as described in the TwistAmp Design Manual^51^. The specificity in-silico assay of primer sets were performed using BLASTn^63^. RPA primers and probes were synthesized by Integrated DNA Technologies (IDT) and Biosearch Technologies Inc., respectively. The *nfo* probes were designed to be located between forward and reverse primers. To adapt the RPA reaction for lateral flow assay, three modifications were added to the probes: 6-carboxyfluorescein or digoxigenin tag at 5′, tetrahydrofuran located around 30 bp of the 5′-end and a polymerase blocking group (C3 spacer) at the 3′-end. *A. marginale, A. ovis,* and *A. phagocytophilum* probes were labeled at 5′ position with fluorescent dye FAM, the GAPDH probe was labeled at 5′ position with DIG (Digoxigenin), and the reverse primers with biotin.

### Artificial positive control (APC)

Two artificial positive controls (APC) were designed, one based on tandem of forward and reverse complement sequences of RPA primers, and the second based on tandem of forward and reverse complement sequences of RPA primers and *nfo* probes targeting *A. marginale, A. ovis, A. phagocytophilum* and GAPDH gene as reported^59^. An APC is a cloneable, synthetic, multi-target, and non-infectious control used for routine application in detection and diagnostics assays^59^. Each sequence was designed and made synthetically then inserted into a pUC57 restriction site (GenScript Inc, USA) (Supplemental Fig. 3A,B).

### RPA optimization

The RPA reaction was performed using the RPA TwistAmp® basic (TwistDx, UK) according to the manufacture’s protocol with modifications. The contribution of betaine (10 μl, 5 M; Thermo Fisher, USA) was evaluated by adding it to the RPA reactions in addition to 3 μl of each DNA sample. The RPA reactions were performed in a dry bath incubator (GeneMate, USA). Incubation was at a constant temperature 35 °C to 40 °C for 20 min or 40 min which was followed by 80 °C for 5 min to deactivate the enzyme complex after DNA amplification. The amplified RPA product was purified by QIAquick PCR Purification Kit (Qiagen, USA) to improve visual discrimination and analyzed by electrophoresis on a 2% agarose gel in 0.5X TAE buffer and SYBR safe (Invitrogen, USA).

The multiplex RPA-*nfo* assay was performed using RPA TwistAmp® nfo kit (TwistDx, UK) according to the manufacture’s protocol with modifications (10 μl betaine). A factorial combination assay to include four volumes of primers (2.1 µl, 1.575 µl, 1.05 µl, and 0.525 µl) and probes (0.6 µl, 0.45 µl, 0.3 µl, and 0.15 µl) was evaluated in order to obtain the optimal combination between internal control and *Anaplasma* species. The RPA incubation followed the same protocol as described above. After amplification, 6 μl of RPA products were mixed with 84 μl of buffer (Abingdon Health, UK), 75 μl of the diluted sample was added to a PCRD Nucleic Acid Detector cassette (Abingdon Health, UK). The results were registered after 15 min.

### Cloning of diagnostic *Anaplasma* species fragments

The three RPA primer sets were adapted for end-point PCR to clone the amplified diagnostic fragments. The PCR amplified products from *A. marginale, A. ovis,* and *A. phagocytophilum* were purified from excised agarose gel bands using QIAquick Gel Extraction Kit (Qiagen, USA). The TOPO TA cloning kit (Invitrogen, USA) was used to clone the three amplified segments of *Anaplasma* according to the manufacturer’s instructions. PCR products were inserted into the pCR’4-TOPO plasmids which were incubated in *Escherichia coli* competent cells following the manufacturer’s protocol. The amplified PCR products carried into transformed bacterial colonies were sequenced to verify whether the *Anaplasma* fragments corresponded to each of the expected *Anaplasma* species in the plasmids. The bacterial plasmids were purified using Plasmid Mini Kit (Qiagen, USA) following the manufacturer’s instructions.

### LC green qPCR of Anaplasma DNA

Total DNA extracted from cattle blood infected with *A. marginale,* sheep blood spiked with *A. ovis* DNA*,* and cattle blood spiked with *A. phagocytophilum* DNA was quantified using LC green quantitative PCR (qPCR). Ten-fold dilutions of previously described plasmid DNA *A. marginale* (8.99 × 10^9^–8.99 × 10^3^ copies/µl)*, A. ovis* (5.04 × 10^9^–5.04 × 10^3^ copies/µl), and *A. phagocytophilum* (4.58 × 10^9^–4.58 × 10^3^ copies/µl), were used to plot standard curves for each of the three *Anaplasma* species DNA quantification. Plasmid DNA concentration was measured by Qubit 4 Fluorometer (Thermo Fisher, USA). The qPCR amplification was carried out in 20 µl final volume containing 10 µl of One Taq Hot Start DNA Polymerase (Biolabs, USA), 0.5 μl of each RPA sense and antisense primer (10 μM), 2 μl of LC green (BioChem, USA), 1 μl of plasmid or DNA sample, 6 μl nuclease-free water. Each reaction was tested in triplicate. The PCR cycling parameters were initial start of 50 °C for 3 min, initial denaturation of 94 °C for 4 min, 40 cycles of denaturation at 95 °C for 20 s, annealing at 62 °C for 20 s, extension at 72 °C for 20 s and final extension final at 72 °C for 4 min. The assays were performed in a Rotor Gene 6000 series (Corbett Research, Qiagen, USA) and the mean of each set of replicates was calculated. The quantity of DNA in each sample was determined by converting the copy number using the formula (amount of DNA (ng) × 6.022 × 10^23^) / (length of DNA (bp) x 10^9^ × 650).

### Limit of detection and specificity of the RPA-basic and RPA-*nfo* assays

The limit of detection of the RPA-basic and RPA-*nfo* assays was assessed using the three cloned plasmids. The limit of detection for the three primer sets was determined using a ten-fold serial dilution of *A. marginale* (8.99 × 10^9^–8.99 × 10^3^ copies/µl)*, A. ovis* (5.04 × 10^9^–5.04 × 10^3^ copies/µl), and *A. phagocytophilum* (4.58 × 10^9^–4.58 × 10^3^ copies/µl). One microliter of each dilution was used as template for RPA-basic and RPA-*nfo* assays.

The specificity of the three RPA primer pairs was tested against the *A. marginale, A. ovis,* and *A. phagocytophilum* DNA reference positive controls. Non-infected tick DNA was used as negative control and NTC (no template control) was also included in all assays. All results were observed by electrophoresis (1X TAE) on agarose gel and lateral flow assay (PCRD).

### Screening of serum and blood samples

Twenty-four cELISA *A. marginale-*positive serum samples, twenty-five blood samples infected with *A. marginale*, and three cell culture preparations infected with *A. phagocytophilum* were screened using published endpoint PCR^44^, qPCR with the optimized RPA primers and the multiplex RPA-*nfo*. All molecular assays were performed using 3 µl of each sample. Twenty microliters of amplified PCR product were electrophoresed and 6 μl of amplified RPA product was mixed with 84 μl of lateral flow buffer, and 75 μl of the diluted sample was loaded to a PCRD.

## Supplementary Information

Supplementary Legends.

Supplementary Information.
