# Supplementary material for: Recombinase polymerase amplification (RPA) with lateral flow detection for three Anaplasma species of importance to livestock health
Source: Sci Rep. 2021 Aug 5;11:15962. doi: 10.1038/s41598-021-95402-y (PMC8342517; doi:10.1038/s41598-021-95402-y)
Supplement: Supplementary file 1 — Supplementary Legends. [file 41598_2021_95402_MOESM1_ESM.docx]

**Supplemental Figure 1**. Artificial Positive Controls (APCs). A) RPA-basic artificial positive control (APC) carrying primer sequences for *A. marginale, A. ovis, A. phagocytophilum,* and the GAPDH housekeeping gene (internal control). B) RPA-*nfo* artificial positive control (APC) carrying primer and probe sequences for *A. marginale, A. ovis, A. phagocytophilum,* and GAPDH housekeeping gene (internal control). Custom synthesized in tandem of RPA forward and reverse complement primer sequences ligated into the multiple cloning sites of pUC57 vector.

**Supplemental Figure 2**. *A. marginale* multiplex RPA-*nfo* using positive serum samples. Lane C, flow-check line; Lane 2, detects FAM/Biotin labelled amplicons (*A. marginale)*; Lane 1, detects DIG/Biotin labelled amplicons (GAPDH gene); 1 – 25, *A. marginale* positive blood samples; APC, Artificial positive control; N, non-template control (water).

**Supplemental Figure 3**. *A. phagocytophilum* multiplex RPA-*nfo* using positive cell culture samples. Lane C, flow-check line; Lane 2, detects FAM/Biotin labelled amplicons (*A. phagocytophilum)*; Lane 1, detects DIG/Biotin labelled amplicons (GAPDH gene) (not present due to cell culture); 26 – 28, *A. phagocytophilum* positive cell culture samples; APC, Artificial positive control; NTC, non-template control (water).
