# Supplementary material for: Recombinase polymerase amplification (RPA) with lateral flow detection for three Anaplasma species of importance to livestock health
Source: Sci Rep. 2021 Aug 5;11:15962. doi: 10.1038/s41598-021-95402-y (PMC8342517; doi:10.1038/s41598-021-95402-y)
Supplement: Supplementary file 2 — Supplementary Information. [file 41598_2021_95402_MOESM2_ESM.pdf]

Supplemental Figure 1

A

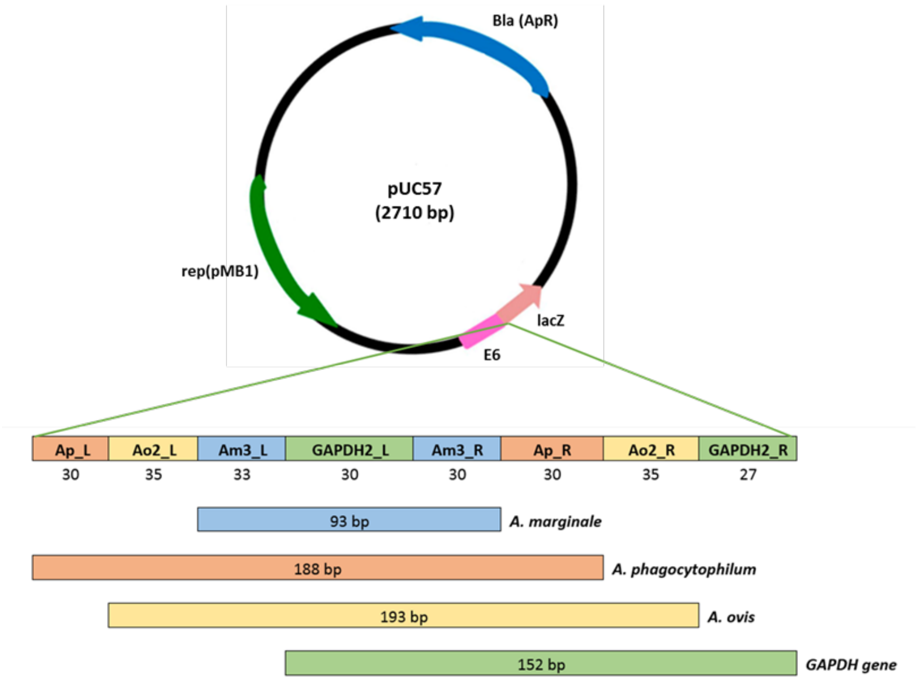

B

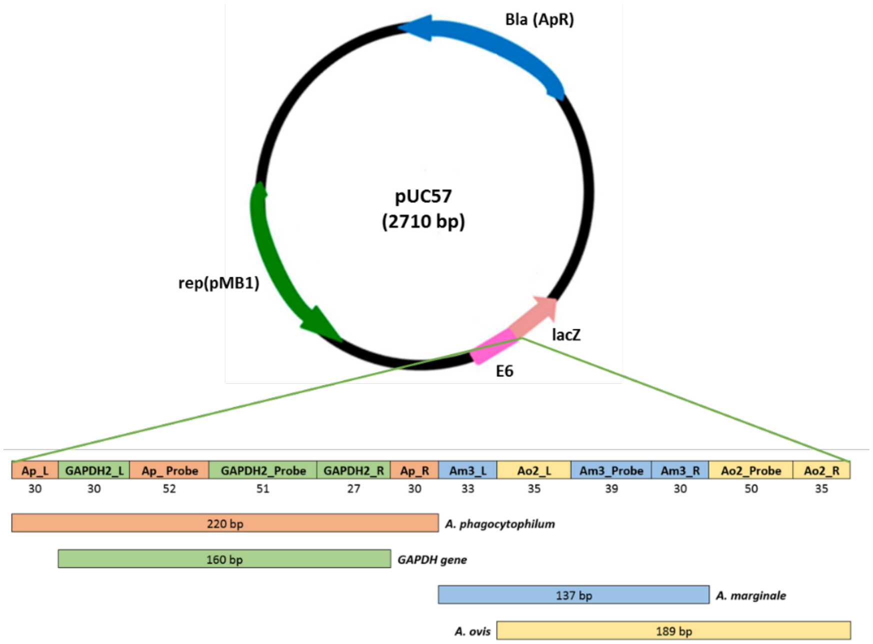

Supplemental Figure 2

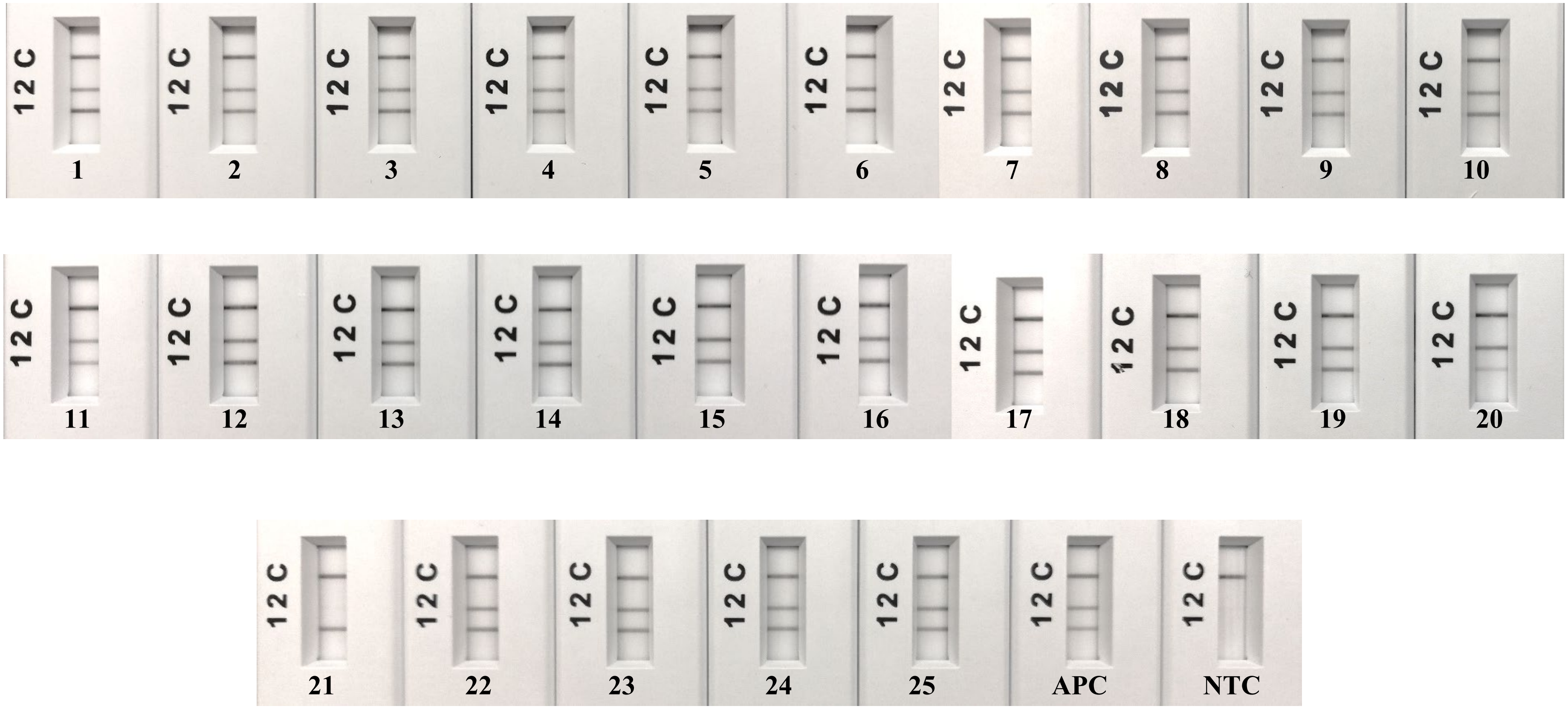

Supplemental Figure 3

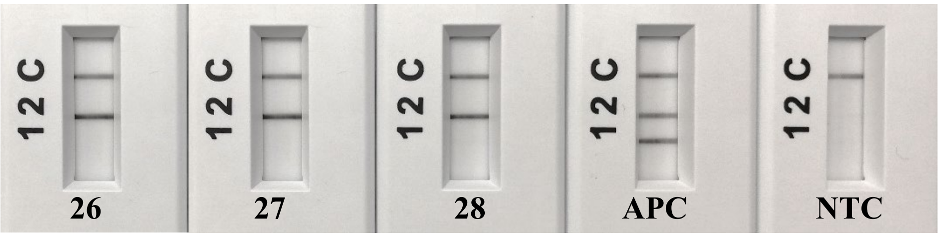

Supplemental Table 1. Endpoint PCR and multiplex lateral flow RPA detection of *A. marginale* from cELISA-positive serum samples provided by Oklahoma State University College of Veterinary Medicine, Stillwater.

**Serum Samples**	***A. marginale* PCR**	**Multiplex RPA-*nfo***	
		**Internal control (Line 1)**	***A. marginale* (Line 2)**
**1**	-	+	-
**2**	-	+	-
**3**	-	+	-
**4**	-	+	+
**5**	-	+	-
**6**	-	+	+
**7**	-	+	+
**8**	-	+	+
**9**	-	+	+
**10**	-	+	+
**11**	-	+	+
**12**	-	+	+
**13**	-	+	+
**14**	-	+	+
**15**	-	+	+
**16**	+	+	+
**17**	-	+	+
**18**	-	+	+
**19**	-	+	+
**20**	-	+	+
**21**	-	+	+
**22**	-	+	+
**23**	-	+	+
**24**	-	+	+
**APC**	NT	+	+
**Am sample**	+	+	+
**NTC**	-	-	-

Supplemental Table 2. Results from Quantitative PCR using optimized RPA primers and multiplex RPA-*nfo* detection of varying prevalence rates of *A. marginale* strains in bovine blood samples and *A. phagocytophilum* from different cell culture samples provided by Oklahoma State University College of Veterinary Medicine, Stillwater.

N°	Cow # / cell line	Sample date / isolate	Blood prevalence	RPA Results		qPCR (RPA primers) (pg/µl)
				Line 1 (IC)	Line 2 (Anaplasma)	
			A. marginale			
**1**	PA407	06/01	16	+	+	5.36
**2**		08/17	0.4	+	+	0.13
**3**		12/14	2	+	+	0.59
**4**		02/12	0	+	+	15.73
**5**	PA412	04/05	21	+	+	12.44
**6**		05/27	2	+	+	2.26
**7**	PA415	04/20	57	+	+	13.6
**8**	PA417	05/26	32	+	+	16.42
**9**		07/06	2	+	+	0.82
**10**		07/30	1	+	+	0.0863
**11**		09/27	0.5	+	+	0.0643
**12**		09/09	9	+	+	0.357
**13**	PA418	05/11	43	+	+	0.546
**14**		07/02	3	+	+	0.128
**15**		07/19	2	+	+	0.0837
			Other *A. marginale* strains			
**16**	PA414	OK	44	+	+	5.533
**17**	PA349	Da*	50	+	+	4.43
**18**	PA413	OK	8	+	+	5.54
**19**	PA420	OK	12	+	+	9.47
**20**	PA428	OK	9	+	+	0.231
**21**	PA430	OK	7	+	-	0
**22**	PA443	OK	20	+	+	1.208
**23**	PA481	VA	35	+	+	13.97
**24**	PA482	OK	9	+	+	11.942
**25**	PA499	OK	8	+	+	3.748
			A. phagocytophilum			
**26**	HL-60		cell culture	-	+	79.35
**27**	Ap74		cell culture	-	+	28.91
**28**	ISE6		cell culture	-	+	24.45

*Da = derived from *Dermacentor albipictus* ticks
